# Barriers and opportunities for the expansion of smoke- and aerosol-free environment policies in Europe

**DOI:** 10.18332/tpc/193977

**Published:** 2024-11-08

**Authors:** Melinda Pénzes, Dolors Carnicer-Pont, Anna Mar López Luque, Helena Koprivnikar, Biljana Kilibarda, Milena Vasic, Adrián González-Marrón, Irene Possenti, Silvano Gallus, Angeliki Lambrou, Efstathios Papachristou, Sotiria Schoretsaniti, Giulia Carreras, Giuseppe Gorini, Esteve Fernández, on behalf of the JATC-2 WP8 Partners

**Affiliations:** 1 Health Services Management Training Centre, Semmelweis University, Budapest, Hungary; 2 National Koranyi Institute of Pulmonology, Budapest, Hungary; 3 Catalan Institute of Oncology, Hospitalet de Llobregat, Barcelona, Spain; 4 Bellvitge Biomedical Research Institute, L’Hospitalet de Llobregat, Barcelona, Spain; 5 Centro de Investigacion Biomedica en Red CIBER, Madrid, Espana; 6 National Institute of Public Health, Ljubljana, Slovenia; 7 Institute of Public Health of Serbia, Belgrade, Serbia; 8 Faculty of Dentistry, Pancevo, Serbia; 9 Group of Evaluation of Health Determinants and Health Policies, Department of Basic Sciences, Universitat Internacional de Catalunya, Barcelona, Spain; 10 Department of Medical Epidemiology, Istituto di Ricerche Farmacologiche Mario Negri IRCCS, Milan, Italy; 11 Directorate of Epidemiology and Prevention of Non-Communicable Diseases and Injuries, National Public Health Organization (NPHO), Athens, Greece; 12 Institute for Cancer Research, Prevention and Clinical Network (ISPRO), Florence, Italy; 13 University of Barcelona, Barcelona, Spain

**Keywords:** e-cigarette vapor, expert opinion, smoke-free policy, secondhand aerosol exposure, secondhand smoke, tobacco control

## Abstract

**INTRODUCTION:**

Comprehensive legislation covering the use of all types of tobacco and nicotine products to provide a smoke- and aerosol-free environment (SAFE) should be part of strategies aimed at phasing out tobacco use. There is a need to identify challenges and opportunities for advancing SAFE policies and their implementation. This study aims to identify barriers and opportunities to extend, enforce, and comply with SAFE policies in Europe.

**METHODS:**

Within the Joint Action on Tobacco Control 2, a cross-sectional expert consultation was launched in 2022. Data obtained through an online questionnaire including closed and open-ended questions on barriers, opportunities, and interference by the tobacco and/or nicotine industry (TNI) on the extension, and compliance with/enforcement of SAFE policies, were analyzed thematically and descriptively.

**RESULTS:**

From 29 European countries, 61 experts (response rate: 55.5%) were included in our sample. The most commonly identified barriers for the extension of SAFE policies were tobacco industry lobbying and funding activities, while the most commonly reported opportunity was extending SAFE policies to specific outdoor public or private places, especially where children are present. In terms of compliance with/enforcement of SAFE policies, the lack of human and financial resources and capacity to monitor/enforce compliance were the most commonly identified barriers, while opportunities included more powerful enforcement authorities with increased capacity. The experts identified greater TNI interference on the extension than on the enforcement of SAFE policies.

**CONCLUSIONS:**

Comprehensive regulation of TNI interference and allocation of human/financial resources for policy enforcement, should be a priority for the extension of SAFE policies in Europe.

## INTRODUCTION

The mortality and morbidity burden from secondhand smoke (SHS) exposure among non-smokers remain significant in the European Union (EU)^1,2^. In addition to the health and economic consequences of SHS exposure, the large number of smoking areas may act as a smoking cue undermining smoking cessation or encouraging relapse to tobacco or nicotine use^3,4^. In recent years, the growing popularity of emerging electronic nicotine or tobacco products, such as various types of e-cigarettes and heated tobacco products (HTPs), has been a challenge in many EU countries^5^. Recent findings warn about the potential health harms of secondhand aerosol (SHA) exposure from both e-cigarettes and HTPs for non-users^6-8^. However, inclusion of such emerging products in national smoke-free policies is inconsistent across Europe^9,10^.

Despite progress towards smoke-free environments in the EU, there are important gaps in the current legislation and its implementation. According to recent European reports, some countries have extended smoke-free policies to more outdoor public places such as restaurant terraces or beaches, and to some private places, such as cars^11-13^. In addition, smoke-free policies are increasingly shifting towards protecting people from involuntary exposure to e-cigarette and HTP aerosol by providing aerosol-free indoor/outdoor and public/private environments^14^. Furthermore, the implementation of smoke-free policies related to traditional smoking products is mostly good, but aerosol-free policies are insufficient for HTPs and even worse for e-cigarettes^12^. Recent European studies summarizing the coverage of and compliance with smoke- and aerosol-free environment (SAFE) policies suggest additional room for the extension of and compliance with such policies^10,12,15^. However, it would also be important to identify the challenges and opportunities for progress with SAFE policies and their implementation.

Within the second European Joint Action on Tobacco Control (JATC–2), Work Package 8 (WP8) aimed to assess the current situation on the implementation of SAFE policies, including outdoor areas and some private settings, and to explore barriers and opportunities to support the extension and enforcement of/compliance with SAFE policies in the EU Member States (MS) and other European countries.

## METHODS

### Study design and procedures

The JATC–2 WP8 team launched a cross-sectional expert consultation study in Europe from June to August 2022. The study employed both quantitative and qualitative approaches, using both closed-ended and open-ended questions to gather in-depth experiences of experts on barriers and opportunities for the expansion and enforcement of SAFE policies through an online questionnaire (implemented in SurveyMonkey). The WP8 team collected the contact details of tobacco control experts from all EU MS and some other European countries (Norway, Serbia, United Kingdom) from lists of own contacts and European organizations involved in tobacco control. Identified experts (n=110) were invited to participate in the study three times by email, and 61 of 110 (55.5%) experts agreed to participate in the consultation and completed the questionnaire. Detailed description of the consultation methodology has been presented elsewhere^16^. Prior to the consultation, all experts were informed about the study in a written electronic document and we obtained written consent from the experts electronically.

### Measures

The study questionnaire was developed by the WP8 team and included questions to identify relevant policies (section 1) and best practices (section 2) on SAFE in Europe, as well as experts’ contact information (country, type of institutional affiliation)^17^. In the current study, we analyzed data related to barriers to the extension of SAFE policies and barriers to the improvement of compliance with/enforcement of SAFE policies, each assessed by a categorical question with response option yes/no and an open-ended question to describe the barriers. We also analyzed data related to opportunities to extend SAFE policies and opportunities to improve compliance with/enforcement of SAFE policies. These questions were structured similarly to the questions used to assess barriers. In addition, experts were asked to indicate the extent to which they thought the tobacco or nicotine industry (TNI) were interfering with the extension and the enforcement of SAFE policies in their countries (response options were: ‘no interference’, ‘small’, ‘moderate’, ‘large’, and ‘very large’ interference; and these response options were collapsed into the categories 'no interference', 'small/moderate', and 'large/very large').

### Analyses

For the four open-ended questions assessing barriers and opportunities for the expansion and improvement of compliance with/enforcement of SAFE policies, a series of thematic analyses were conducted using subjective coding systems by three WP8 researchers. The responses were categorized thematically, but this classification resulted in a high number of categories (n=11–15). Therefore, the high number of thematic categories was collapsed into broader thematic categories (n=5–6). The number of identified themes on barriers and opportunities for the expansion and improvement of compliance with/enforcement of SAFE policies was calculated per country. Descriptive statistics, including frequencies and cross-tabulations, were calculated for all broader thematic categories. Opinions of TNI interference with the extension or enforcement of SAFE policies were described overall and by institutional affiliation of respondents using frequencies and percentages. Quantitative analyses were conducted using IBM SPSS version 28.0.

## RESULTS

### Characteristics of the sample

Of the 110 invited experts, 61 experts from 29 European countries responded the consultation. The number of respondents per country is shown in Table 1. Regarding the institutional background of the responding experts, 59.0% were affiliated to government or public institutions, 26.2% to non-governmental organizations (NGOs) or tobacco control/public health national societies, and 14.8% to universities.

**Table 1:** Number of responding experts and number of identified barriers and opportunities for the expansion and enforcement of, and compliance with smoke- and aerosol-free environment policies, by country

*Countries*	*Experts who responded*	*Identified barriers for the expansion of SAFE policies*	*Identified barriers to the compliance with/enforcement of SAFE policies*	*Identified opportunities for the expansion of SAFE policies*	*Identified opportunities to the compliance with/enforcement of SAFE policies*	*Number of experts who identified TNI interference with the expansion of SAFE policies*	*Number of experts who identified TNI interference with the enforcement of SAFE policies*
Austria	3	3	2	2	4	3	3
Belgium	3	2	3	2	1	2	1
Croatia	2	0	0	0	0	1	1
Cyprus	3	2	1	0	0	1	1
Czechia	3	3	3	3	3	3	2
Denmark	2	4	0	3	1	2	1
Estonia	3	1	1	2	1	2	1
Finland	1	2	1	1	0	1	1
France	2	3	5	3	2	2	2
Germany	2	3	2	2	2	2	1
Greece	2	1	0	1	1	1	1
Hungary	3	0	0	0	4	3	0
Ireland	2	2	2	3	3	1	0
Italy	2	1	3	3	1	1	1
Latvia	2	2	0	0	0	1	1
Lithuania	1	1	3	1	2	1	1
Luxemburg	2	1	1	1	0	2	2
Malta	1	2	1	1	1	1	1
Netherlands	6	5	3	3	4	3	3
Norway	2	3	2	3	1	2	1
Poland	1	1	1	1	1	1	1
Portugal	1	1	1	0	0	1	1
Romania	1	2	3	1	2	1	1
Serbia	1	0	0	0	0	0	0
Slovakia	1	0	0	0	0	1	1
Slovenia	4	6	6	6	5	4	4
Spain	1	2	2	1	1	1	1
Sweden	2	1	2	2	2	2	2
UK	2	3	1	2	3	2	2
Total	61	57	49	47	45	48	40

SAFE: smoke- and aerosol-free environment. TNI: tobacco and/or nicotine industry.

### Barriers to the expansion of SAFE policies

Forty-two experts (68.8%) reported 57 barriers to the expansion of SAFE policies (Table 1), while 9 experts identified no barrier. Most of the barriers identified were related to tobacco industry lobbying and funding activities, including lobbying of parliamentarians, civil servants, health professionals or members of small businesses, and/or funding of ‘smoke-free’ and ‘harm reduction’ campaigns (e.g. in social media), as well as funding of events to promote social acceptance of HTPs in indoor environments (Table 2). In addition, experts mentioned the reluctance and low commitment of the governments and authorities to expand SAFE policies. They also reported a lack of development and implementation of legislation for SAFE outdoor places, a lack of monitoring and a lack of sales regulation. One of the barriers reported was claims of specific settings against the expansion. These settings were the hospitality and tourism sector, the small business sector, and private homes. In all, 11.5% of the responding experts mentioned misinformation about emerging nicotine and tobacco products as a barrier to the expansion of SAFE. That is, they perceived that both the public and health professionals are misinformed or lack information about HTPs and e-cigarettes, and many of them believe that there is a lack of evidence on the harmful health effects of these products. Lack of capacity and public/professional support to enforce SAFE policies and some other barriers such as non-stigmatization of people who smoke, were also mentioned.

**Table 2:** Thematic categories of identified barriers and opportunities for the expansion and enforcement of, and compliance with smoke- and aerosol-free environment policies (N=61)

*Thematic categories*	*Experts who responded*	
	*n*	*%*
**Expansion of SAFE policies**		
**Barriers**		
Tobacco industry lobby and funding activities	15	24.6
Reluctance and low commitment of government and competent authorities for the expansion	13	21.3
Claims of specific settings against the expansion	13	21.3
Misinformation about novel nicotine and tobacco products	7	11.5
Lack of capacity and public or professional support for enforcing	6	9.8
Other	4	6.6
No barrier identified	9	14.8
**Opportunities**		
Expanding SAFE policies to certain outdoor places	17	27.9
Supporting attitude of citizens/politicians/governmental organizations/NGOs towards SAFE policies	8	13.1
Other	8	13.1
National ‘smoke-free’ or ‘smoke-free generation’ strategy	6	9.8
Local campaigns and education for understanding SAFE policies	5	8.2
Extension of SAFE legislation for emerging nicotine and tobacco products	3	4.9
No opportunity identified	10	16.4
**Compliance with/enforcement of SAFE policies**		
**Barriers**		
Lack of human/financial capacity for supervision/enforcement	20	32.8
Reluctance and low commitment of government and authorities to the improvement of compliance with or enforcement of SAFE policies	11	18.0
Lack of training/education for authorities and/or the public	9	14.8
Other	6	9.8
Tobacco industry lobby and funding	4	6.6
No barrier identified	17	27.9
**Opportunities**		
More powerful enforcement authorities with increased capacities	14	23.0
Public education, awareness raising/communication campaign	10	16.4
Other	9	14.8
Comprehensive SAFE policies should be expanded for other indoor/outdoor areas	5	8.2
Funding for prevention/communication campaigns, and monitoring	5	8.2
No opportunity identified	12	19.7

SAFE: smoke- and aerosol-free environment. Percentages do not sum 100% due to multiple reporting of policies per expert.

### Opportunities for the expansion of SAFE policies

Thirty-nine experts (63.9%) identified 47 opportunities for extending SAFE policies (Table 1), while 10 experts identified no opportunity. More than a quarter (27.9%) of the experts believed that there would be opportunities to extend SAFE policies to certain outdoor places, such as beaches, parks, crowded places, places where children are present, hospitality venues, balconies of private homes, and cars (Table 2). Improving the supportive attitudes of citizens, politicians, governmental organizations, and NGOs towards SAFE policies was also reported as an opportunity. Several experts mentioned that ongoing or recently launched national ‘smoke-free’ or ‘smoke-free generation’ strategies, as well as local campaigns and education for the general population to understand SAFE policies, could be further opportunities for the expansion. Eight respondents also mentioned a wide range of other opportunities including transparency of industrial financial operations, funding for cessation services or enforcement of SAFE policies, and the imposition of significant fine as a deterrent. Finally, three experts supported the extension of SAFE legislation to emerging nicotine and tobacco products, while two experts opposed the extension of SAFE policies to these products.

### Barriers to the compliance with/enforcement of SAFE policies

About half of the respondents (n=32; 52.5%) identified 49 barriers to the compliance with or enforcement of SAFE policies (Table 1), while 17 experts identified no barrier. Nearly a third of experts (32.8%) reported the lack of human and financial resources and capacity to effectively monitor compliance with SAFE policies and apply sanctions where necessary as the major barrier (Table 2). In addition, the reluctance and low commitment of governments and authorities to improve compliance with or enforcement of SAFE policies was also frequently reported, including the lack of comprehensive and clear legislation on SAFE, and the lack of internal institutional guidelines or legal frameworks for the enforcement of SAFE policies. Other barriers identified were the lack of training for competent authority staff to communicate the importance of SAFE policies and the lack of education either on the health harms of outdoor SHS/SHA exposure or on possible behavior change strategies. Experts indicated that tobacco industry lobbying and funding toward parliamentarians, civil servants, small businesses, and health professionals could also lead to lower compliance with SAFE policies. Some other barriers were less frequently mentioned, such as low public support, lack of differentiation between smokers and non-smokers in health insurance premiums, or difficulties in extending SAFE policies to private homes.

### Opportunities for compliance with/enforcement of SAFE policies

More than half of the respondents (n=35; 57.4%) identified 45 opportunities to improve the compliance with or enforcement of SAFE policies (Table 1), while 12 experts identified no opportunity. Most of them recommended that competent authorities should have greater capacity to enforce SAFE policies (Table 2). Several experts also mentioned the importance of public education, awareness campaigns and regular communication on the importance of SAFE policies. In addition, funding opportunities for nicotine and tobacco prevention and continuous monitoring would also be needed. Five experts highlighted the opportunity to extend SAFE policies to additional indoor and outdoor settings. Several other possible opportunities were reported, such as increasing taxes or implementing and enforcing tobacco advertisement, promotion and sponsorship measures for emerging tobacco and nicotine products. In addition, resolving conflicting positions of health and finance ministries, promoting cultural changes towards SAFE, and controlling TNI interference, particularly for the expansion of HTPs, were reported by the experts.

### Interference of tobacco or nicotine industries and their allies (TNI) with the expansion and enforcement of SAFE policies

Of the 61 experts, 48 (78.7%) indicated that TNI interfered to some extent with the extension, and 40 (65.6%) reported any TNI interference with the enforcement of SAFE policies in their countries (Table 3). More than a third of the participating experts believed that TNI interfered largely or very largely with the expansion of SAFE policies. In contrast, most of the experts perceived that there was small/moderate interference with enforcement. Experts with university affiliations were more likely to report large/very large TNI interference with the extension of SAFE policies compared to experts with NGOs/societies, while respondents from governmental/public institutions were even less likely to do so. Large/very large TNI interference in the enforcement of SAFE policies was rarely reported, especially by experts from governmental/public institutions.

**Table 3:** Perceived extent of tobacco or nicotine industry interference on SAFE policies, overall and by institutional affiliation of responding experts (N=61)

	*Perceived extent of interference*			
	*No response n (%)*	*No interference n (%)*	*Small/moderate n (%)*	*Large/very large n (%)*
**TNI interference with the extension of SAFE policies**				
Total	12 (19.7)	1 (1.6)	26 (42.6)	22 (36.1)
Governmental/Public institutions	7 (19.4)	1 (2.8)	19 (52.8)	9 (25.0)
NGOs/Societies	3 (18.8)	0 (0.0)	6 (37.5)	7 (43.8)
Universities	2 (22.2)	0 (0.0)	1 (11.1)	6 (66.7)
**TNI interference with the enforcement of SAFE policies**				
Total	12 (19.7)	9 (14.8)	32 (52.5)	8 (13.1)
Governmental/Public institutions	7 (19.4)	7 (19.4)	19 (52.8)	3 (8.3)
NGOs/Societies	3 (18.8)	2 (12.5)	9 (56.3)	2 (12.5)
Universities	2 (22.2)	0 (0.0)	4 (44.4)	3 (33.3)

SAFE: smoke- and aerosol-free environment. TNI: tobacco and/or nicotine industry and their allies.

## DISCUSSION

Our study highlights that there are numerous barriers for the extension and enforcement of, and compliance with SAFE policies in Europe. However, promising opportunities were also identified. Based on the opinions of tobacco control experts, especially those with NGO or university affiliations, the TNI lobby was the most important barrier to the extension of SAFE policies. However, our findings suggest that the extent of TNI interference in the enforcement is much lower than in the extension of SAFE policies, perhaps simply because the enforcement is not effectively done. Thus, it seems that the tobacco industry is still more active in lobbying against the enactment or even planned extension of SAFE legislation than in obstructing compliance or enforcement of implemented policies. However, if enforcement of such policies were much stronger, TNI interference against compliance or enforcement would likely to be more active. Our results are consistent with recent evidence that TNI interference remains the biggest obstacle to European tobacco control policy-making and represents a serious problem in almost all European countries^18,19^. TNI interference to delay smoke-free legislation through various claims, political lobbying and/or donations, and finding legislative loopholes for their interests, has a decades-long history^20^. One of the solutions to this problem is the complete implementation of the WHO Framework Convention on Tobacco Control (FCTC) Article 5.3 and its guidelines about protection of health policies from commercial and other vested interests of the tobacco industry in the legislation of European countries, including the EU Tobacco Products Directive^21^. However, no European government has currently fully implemented Article 5.3. In addition, the majority of countries ignore TNI interference in national tobacco control decision-making, and have not developed a code of conduct for public or government officials that directly refers to Article 5.3^11,19^. Similarly to previous studies, the reluctance of governments and competent authorities as well as the claims of the hospitality and tourism sector against the extension of SAFE, were commonly identified barriers in the present study^12,22^.

On the other hand, the results show that the most important opportunity to extend SAFE policies would be to apply them to certain outdoor public places, such as beaches, parks, crowded places, quasi-outdoor public places, such as hospitality venues, open private places like balconies, enclosed private spaces such as cars, and, in general, places where children are present. Implementation of extended policies covering different types of outdoor public or private spaces is rare in Europe, despite being encouraged by Article 8 of the WHO FCTC ^12,20,23^. In addition to SAFE policies at the national or supra-national level, complementary regulatory measures led by sub-national jurisdictions (municipalities, provinces or regions/states) should also be considered in such an extension^23^. Recent population surveys have found that non-smokers and former smokers are particularly supportive for extending smoke-free and aerosol-free policies in public and private places, especially where children are frequently exposed to tobacco smoke, and besides, even smokers are moderately supportive of such extensions^5,9,24,25^. Therefore, framing SAFE policies as a child health, child rights, and human rights issue seems promising^23,24,26^ and it is also in alignment with the recent landmark decision on human rights and tobacco control by the Tenth Session of the Conference of Parties to the WHO FCTC^27^. This decision could encourage countries to integrate human rights principles when implementing tobacco control policies and calls for collaboration between WHO FCTC and United Nations human rights bodies^28^. This may increase public awareness of and support for extended SAFE policies in the population.

Promoting awareness that underscores the imperative of shielding youth from any and all exposure to tobacco and nicotine may embolden many communities to advocate for comprehensive SAFE policies^26^. Thus, public pressure could urge governments to act at the legislative level, taking into account children’s and human rights issues. Experts in our study also highlighted the importance of investing in campaigning and education for SAFE to ensure broad public and stakeholder support for it. Such attitude shaping activities may be valuable in overcoming the reluctance of competent authorities to adopt SAFE policies. Furthermore, addressing some barriers, such as claims of specific settings or misinformation about emerging products, also requires prior education and attitude shaping about these products and the relevance of SAFE, e.g. for stakeholders in the hospitality and tourism sector, in order to successfully achieve the extension of SAFE and counteract TNI interference.

Only a few experts from some countries suggested extending SAFE legislation for emerging nicotine and tobacco products such as e-cigarettes and HTPs. Many European countries have recently extended smoke-free policies to these emerging products, but the coverage of complete or partial bans on their use varies widely across countries and by type of setting^10,13^. Our findings suggest that most of the responding experts would prioritize the extension of SAFE policies to a broader range of outdoor or private settings, while probably not advocating a complete ban on emerging products. However, balancing the coverage of settings and product types during planning and decision-making on the extension of SAFE policies would be needed in order to ensure that the society and its different sectors understand and accept the gradual introduction and implementation of measures to curb the tobacco epidemic.

Our findings revealed serious gaps in the human and financial capacity to enforce SAFE policies across Europe. The lack of capacity to enforce smokefree policies appears to have remained unchanged since the last EU-wide survey^12^. Successful legislative implementation of SAFE policies does not necessarily lead to full compliance and adequate enforcement^12,22^. There would be a general need for government involvement and commitment to clear, comprehensive guidelines and expectations for enforcement^20,22^, which was also mentioned by experts. Stakeholders responsible for decision making (e.g. policymakers in government) and policy enforcement (e.g. public health authorities, healthcare service management, hospitality sector leaders) should understand the importance of SAFE policies. Therefore, shaping stakeholders’ attitudes towards SAFE by educating them on the individual, population, economic, and environmental impacts of SHS and SHA, would be critical to the adoption of legal measures and to improve overall compliance and enforcement at all levels^20,22^. Investing in public education and awareness raising campaigns by using culturally and community-specific communication channels was identified as a way to overcome low compliance with SAFE policies. In addition, some experts complained about TNI interference, which affects compliance with and enforcement of SAFE policies. To prevent such interference, government collaboration with civil societies and NGOs to mitigate TNI tactics, could be a viable and increasingly applied solution^20,22^.

To overcome the lack of monitoring or enforcement capacity, the use of innovative human and digital resources to improve the enforcement of SAFE policies should be urgently considered. For example, the use of digital solutions, such as smartphone apps, for enforcement, combined with voluntary reporting of violations by citizens or a group of people in specific settings, could provide an active surveillance strategy to monitor compliance with SAFE policies^28,29^. Artificial intelligence-based monitoring of indoor and outdoor compliance with smoke-free and aerosol-free regulations could be another novel solution^30^. In addition, such solutions could facilitate targeted inspections and enforcement by the national competent authority, even visualize non-compliance in real time for both the government authorities and for the public, and integrate educational elements to disseminate the importance of SAFE^28,29^.

Funding to ensure monitoring and enforcement of SAFE policies and continuous public awareness raising campaigns would also be crucial. However, overall funding for tobacco control is critically low in most European countries^11^. All WHO FCTC measures and their implementation guidelines, including Article 8, require effective and sustainable funding to be successfully implemented in the long-term. In order to curb the harmful effects of tobacco and nicotine use on the European population, it would be timely to exploit well-known and possible innovative revenue resource solutions for tobacco control through the implementation of local, national, and collective EU-wide regulatory changes^31^.

Interestingly, a significant minority of experts (about 15%) did not identify any barriers or opportunities for the extension of SAFE policies, while an even higher proportion of experts (20–28%) identified for the compliance with/enforcement of SAFE policies. These experts were mainly from Central Eastern European countries. Exploring the reasons for ‘no’ answers in these countries would be valuable in future studies, as perhaps slightly different approaches would be needed to successfully advance SAFE policies than in Western European countries.

### Strengths and limitations

Some limitations of our study need to be addressed. Firstly, almost half of the invited experts did not participate in the consultation. Therefore, conclusions from our findings should be drawn with caution due to potential selection bias. However, we found no pattern in the acceptance or rejection of participation.

In addition, there were some countries (Croatia, Serbia, and Slovakia) where experts did not report any barriers or opportunities for the expansion or compliance with/enforcement of SAFE policies. Respondent bias may have existed as experts’ different institutional affiliations and professional experiences may have influenced the content of their responses and their commitment to SAFE policies. However, the consultation has several strengths. The timing of the consultation allowed for a quasi-follow-up assessment of expert opinion conducted in 2020–2021 on these issues^12^ and to identify the direction of change in the implementation of clean air policies in Europe. Moreover, the use of open-ended questions in the consultation and the detailed responses to these questions provided a broader insight into the nature and extent of barriers and opportunities per country. Finally, potential conflicts of interests with TNI were assessed during the consultation, thus preventing additional responding bias.

## CONCLUSIONS

Our study confirmed that there is still room for progress in SAFE policies in Europe. However, comprehensive regulation to hamper TNI interference and allocation of human/financial resources for policy enforcement should be a priority for the extension of SAFE policies in Europe. As a next step, it is essential to reconsider sustainable funding for tobacco control, which could support additional preventive measures such as education, communication campaigns, and monitoring.

## Data Availability

The data supporting this research are available from the authors on reasonable request.
